# Sodium butyrate reduces high-fat diet-induced non-alcoholic steatohepatitis through upregulation of hepatic GLP-1R expression

**DOI:** 10.1038/s12276-018-0183-1

**Published:** 2018-12-03

**Authors:** Da Zhou, Yuan-Wen Chen, Ze-Hua Zhao, Rui-Xu Yang, Feng-Zhi Xin, Xiao-Lin Liu, Qin Pan, Huiping Zhou, Jian-Gao Fan

**Affiliations:** 1 Center for Fatty Liver, Department of Gastroenterology, Xinhua Hospital Affiliated to Shanghai Jiao Tong University School of Medicine, 200092 Shanghai, China; 2 Department of Gastroenterology, Zhongshan Hospital of Fudan University, 200032 Shanghai, China; 3 Department of Microbiology and Immunology, Department of Internal Medicine/GI Division, McGuire VA Medical Center, Richmond, VA 23298 USA; 4 Shanghai Key Lab of Pediatric Gastroenterology and Nutrition, Xinhua Hospital Affiliated to Shanghai Jiao Tong University School of Medicine, 200092 Shanghai, China

## Abstract

Glucagon-like peptide-1 (GLP-1) has a broad spectrum of biological activity by regulating metabolic processes via both the direct activation of the class B family of G protein-coupled receptors and indirect nonreceptor-mediated pathways. GLP-1 receptor (GLP-1R) agonists have significant therapeutic effects on non-alcoholic fatty liver disease (NAFLD) and steatohepatitis (NASH) in animal models. However, clinical studies indicated that GLP-1 treatment had little effect on hepatic steatosis in some NAFLD patients, suggesting that GLP-1 resistance may occur in these patients. It is well-known that the gut metabolite sodium butyrate (NaB) could promote GLP-1 secretion from intestinal L cells. However, it is unclear whether NaB improves hepatic GLP-1 responsiveness in NAFLD. In the current study, we showed that the serum GLP-1 levels of NAFLD patients were similar to those of normal controls, but hepatic GLP-1R expression was significantly downregulated in NAFLD patients. Similarly, in the NAFLD mouse model, mice fed with a high-fat diet showed reduced hepatic GLP-1R expression, which was reversed by NaB treatment and accompanied by markedly alleviated liver steatosis. In addition, NaB treatment also upregulated the hepatic p-AMPK/p-ACC and insulin receptor/insulin receptor substrate-1 expression levels. Furthermore, NaB-enhanced GLP-1R expression in HepG2 cells by inhibiting histone deacetylase-2 independent of GPR43/GPR109a. These results indicate that NaB is able to prevent the progression of NAFL to NASH via promoting hepatic GLP-1R expression. NaB is a GLP-1 sensitizer and represents a potential therapeutic adjuvant to prevent NAFL progression to NASH.

## Fatty liver disease: A gutsy way to prevent disease progression

A treatment for non-alcoholic fatty liver disease that incorporates a metabolite found in the gut could prevent progression to a more serious liver condition. Drugs that enhance the activity of glucagon-like peptide-1 (GLP-1), a protein involved in regulating metabolic processes, have shown promise in targeting non-alcoholic fatty liver disease and the more serious condition, steatohepatitis. However, some patients appear resistant to treatment. Jian-Gao Fan at Shanghai Jiao Tong University in China, Huiping Zhou at McGuire VA Medical Center in Richmond, USA, and co-workers demonstrated that a gut metabolite called sodium butyrate may help encourage responsiveness to GLP-1 treatment. The team found that liver GLP-1R expression was considerably reduced in patients with liver disease compared with healthy controls. Experiments on mouse models showed that treatment incorporating sodium butyrate improved GLP-1R levels and reduced fatty liver deposits.

## Introduction

Non-alcoholic fatty liver disease (NAFLD) is emerging as the most common chronic liver disease globally^1,2^. NAFLD comprises a group of diseases that include non-alcoholic fatty liver (NAFL), non-alcoholic steatohepatitis (NASH) and related cirrhosis, and it is often associated with insulin resistance and metabolic syndrome. NASH is a more severe type of NAFLD, and no effective treatment is available^3^.

The short-chain fatty acid (SCFA) butyrate, which is produced by the microbial fermentation of dietary fiber in the large intestine, has been shown to have multiple beneficial effects in mammals^4–9^. Our recent studies reported that sodium butyrate (NaB) reduced high-fat diet (HFD)-induced gut microbiota dysbiosis and endotoxemia^10^ and prevented HFD-induced steatohepatitis by modulating the immune response in the gut and liver^11^. However, the underlying cellular/molecular mechanisms remain unclear.

Evidence shows that NaB could promote glucagon-like peptide-1 (GLP-1) secretion from the intestinal L cells^12^. GLP-1 is reported to exert important functions in the management of type 2 diabetes (T2D) and obesity, including regulation of glucose homeostasis, gastric motility and food intake. Specifically, this hormone exerts its effects via activating the class B G protein-coupled receptor (GPR) GLP-1R^13^. Cumulative evidence shows that GLP-1 exerts direct and beneficial effects on hepatocytes to protect against the progression of NAFL to NASH, suggesting that GLP-1 could be a novel therapy for NASH^14–17^. However, some clinical practices showed that GLP-1-based therapies have little effect on hepatic steatosis or fibrosis in some patients with T2D and NAFLD^18,19^. For example, a previous work reported that some patients with T2D did not respond to GLP-1 treatment^20^. However, it remains unknown whether hepatic GLP-1 resistance contributes to the poor treatment efficiency of GLP-1 in these patients.

In the current study, we hypothesized that the loss of responsiveness to GLP-1 contributes to NAFLD disease progression and the beneficial effect of NaB on NAFLD is partially due to its improvement of hepatic GLP-1 sensitivity.

## Materials and methods

### Patients and specimens

Liver specimens were obtained from 10 adult patients who underwent liver biopsies and met the diagnostic criteria for NAFL or NASH^21^ and eight normal controls from surgeries due to accidents between January 2016 and December 2016; all specimens were collected at Xinhua Hospital (Shanghai, China). Serum specimens from 40 biopsy-proven or ultrasound-proven NAFLD adult patients and 50 adult normal controls were collected during the same period at Xinhua Hospital. The ages and genders between the NAFLD and normal control groups exhibited no differences. All subjects with the following conditions were excluded from the study: history of alcohol consumption, cancer, diabetes mellitus, or any other diseases associated with the liver. Peripheral venous blood samples were drawn after overnight fasting for 12 h. This study was approved by the Human Ethics Committee of Xinhua hospital affiliated with Shanghai Jiao Tong University School of Medicine, and written informed consent was obtained from each participant.

### Animal experiments

Specific pathogen-free male C57BL/6 mice (SLAC Laboratory Animal Co., Ltd., Shanghai, China) were used. A total of 45 mice were randomly assigned to three groups. The control group was fed a standard diet for 16 weeks, and both the HFD group and the intervention group (HFD + NaB) were fed a high-lard-fat and high-cholesterol diet (88% standard diet, 10% lard, and 2% cholesterol, SLAC Laboratory Animal Co., Ltd.) for 16 weeks. After 8 weeks of the HFD, the HFD + NaB group underwent daily intragastric administration of NaB at 200 mg/kg body weight for 8 weeks, while the HFD group received the same amount of saline daily for 8 weeks. Three randomly selected mice from each group were euthanized before intervention at 8 weeks. Body weights and food consumption levels were recorded weekly. After 8 weeks of intervention, the mice were fasted for 12 h, and blood or tissue samples were collected.

All animal experiments were approved by the Institutional Animal Care and Use Committee of Xinhua Hospital affiliated with Shanghai Jiao Tong University School of Medicine and were conducted in accordance with Guide for the Care and Use of Laboratory Animals (National Research Council 1996, USA).

### Cell culture and treatment

The human hepatoma cell line (HepG2) was obtained from the American Type Culture Collection (Manassas, VA, USA). These cells were cultured in Dulbecco’s modified Eagle’s medium supplemented with 10% fetal bovine serum (Gibco BRL, USA) and were maintained in a humidified incubator containing an atmosphere of 5% CO_2_ at 37 °C. To establish a cell model of fat overloading, palmitic (C16:0) and oleic (C18:1) acids (free fatty acids, FFAs) were added into the culture solution at a final concentration of 0.5 mM at a 1:2 ratio^22,23^.

*Experiment 1*: Cells were treated with increasing concentrations of FFAs (0.25, 0.5, and 1.0 mM), lipopolysaccharides or tumor necrosis factor-α for 24 h or were treated with FFAs (0.5 mM) at different times (12, 24, 36, and 48 h).

*Experiment 2*: Cells were treated with increasing concentrations of NaB (1, 2, 5, 10 mM) or histone deacetylase (HDAC) inhibitors (CI-994, HDAC1 inhibitor, 5 μM; Santacruzamate A, HDAC2 inhibitor, 1 nM; RGFP966, HDAC3 inhibitor, 1 μM; PCI-34051, HDAC8 inhibitor, 100 nM; Trichostatin A (TSA), 10 nM) or vehicle for 24 h.

*Experiment 3*: Cells were treated with NaB (5 mM) or vehicle for 24 h; after 24 h, the media was replaced and the cells were treated with GLP-1 (200 nM, Liraglutide, Novo Nordisk, Denmark) for another 24 h.

### Inhibition of GPR43/GPR109a expression by RNA interference

Human G-protein-coupled receptor 43 (GPR43) or GPR109a siRNA or negative control (NC) siRNA was mixed with the Lipofectamine 2000 transfection reagent (Invitrogen, California, USA) (Table S1). Then, GPR43 (50 nM), GPR109a (50 nM), and NC siRNA (50 nM) were delivered into the HepG2 cells (~40% confluent density) and cultured in Opti-MEM (Gibco) for 6 h, after which the transfection mixture was replaced with normal medium. After 24 h of transfection, the cells were cotreated with FFAs and NaB (5 mM) for an additional 24 h before harvest. All siRNAs were purchased from GenePharma (Shanghai, China).

### Serum biochemical index detection

Serum alanine aminotransferase (ALT), aspartate aminotransferase (AST), and fasting blood glucose were measured using an automated analyzer (Sysmex CHEMIX-180, Japan). Insulin (Rat/Mouse Insulin ELISA Kit, Merck-Millipore) and GLP-1 (Glucagon Quantikine ELISA Kit, R&D Systems) in the serum were measured using an enzyme-linked immunosorbent assay. A homeostatic model assessment of insulin resistance (HOMA-IR) and the insulin sensitive index (ISI) were calculated.

### Intracellular and intrahepatic triglyceride (TG) measurement

Intracellular TG and intrahepatic TG levels were measured using a TG assay kit (Applygen Technologies Inc., Beijing, China). Samples and standards were then processed according to the manufacturer’s instructions. The final concentrations of TGs and cholesterol were corrected for protein content^11^.

### Histological examination of the liver

Human or mouse liver tissues were stained with hematoxylin and eosin (HE). The nonalcoholic fatty liver activity scores (NAS) were graded^24^. Standard pieces of liver tissues from mice were embedded in an optimal cutting temperature gel and stained with Oil Red O solution^25^. Images were captured using a microscope (Leica DMI3000B, USA).

### Immunohistochemistry

Briefly, paraffin-embedded liver tissues were deparaffinized with xylene and washed with graded ethanol. The inactivation of endogenous peroxidase was achieved using 3% H_2_O_2_ for 20 min. These tissue sections were washed with phosphate-buffered saline (PBS) and incubated overnight with anti-GLP-1R (1:200, NBP1-97308, Novus Biologicals, USA) at 4 °C. Staining with 3,3-di-amino-benzidine (DAB, Santa Cruz, USA) was used for visualization and images were captured using a microscope (Leica DMI3000B, USA). The relative positive areas were quantified with ImageJ (1.51j8, USA)^11^.

### Western blot analysis

Tissues or cells were lysed in ice-cold radio-immunoprecipitation assay (P0013B, RIPA) buffer containing protease and phosphatase inhibitors (phenylmethylsulfonyl fluoride, ST506, Beyotime, Shanghai, China). Total protein was measured using the bicinchoninic acid protein assay (P0010, BCA, Beyotime, Shanghai, China). Western blot analysis was performed as previously described^23^, and the antibodies were applied at concentrations according to the manufacturer’s instructions. Immune complexes were detected using the Immobilon Western Chemiluminescent HRP Substrate (WBKLS0050, Millipore Corporation, Billerica, MA). Actin served as the loading control. Bands were quantified using Image Lab Version 2.0.1 (Bio-Rad, Hercules, CA).

Anti-Actin (AA128, Beyotime), anti-GPR109a (ab81825, Abcam, USA), anti-GPR43 (DF2746, Affinity Biosciences, USA), anti-H3 (anti-total histone H3, 4499, Cell Signaling Technology, CST, USA), anti-Ace-H3 (anti-pan-acetyl histone H3, 61637, Active Motif, USA), anti-p-AMPK (2535, adenosine monophosphate activated protein kinase, CST, USA), anti-p-ACC (11818, p-acetyl-CoA carboxylase, CST), anti-IR (anti-insulin receptor, ab5500, Abcam), anti-IRS-1 (anti-insulin receptor substrate-1, ab46800, Abcam), and anti-GLP-1R (NBP1-97308, Novus Biologicals, USA) were used in our study.

### Statistical analysis

The data are expressed as the means with the standard error of the mean (SEM). Comparisons were performed using one-way analysis of variance (ANOVA), and Spearman’s correlations were calculated in GraphPad Prism 5 software (version 6.01). Post hoc Student–Newman–Keuls analyses were performed when >2 groups were present. The multiple linear regression analysis was performed using IBM SPSS statistics software (version 19). A *P* < 0.05 was considered statistically significant.

## Results

### GLP-1R was downregulated in the livers of NAFLD patients

**Fig. 1:**
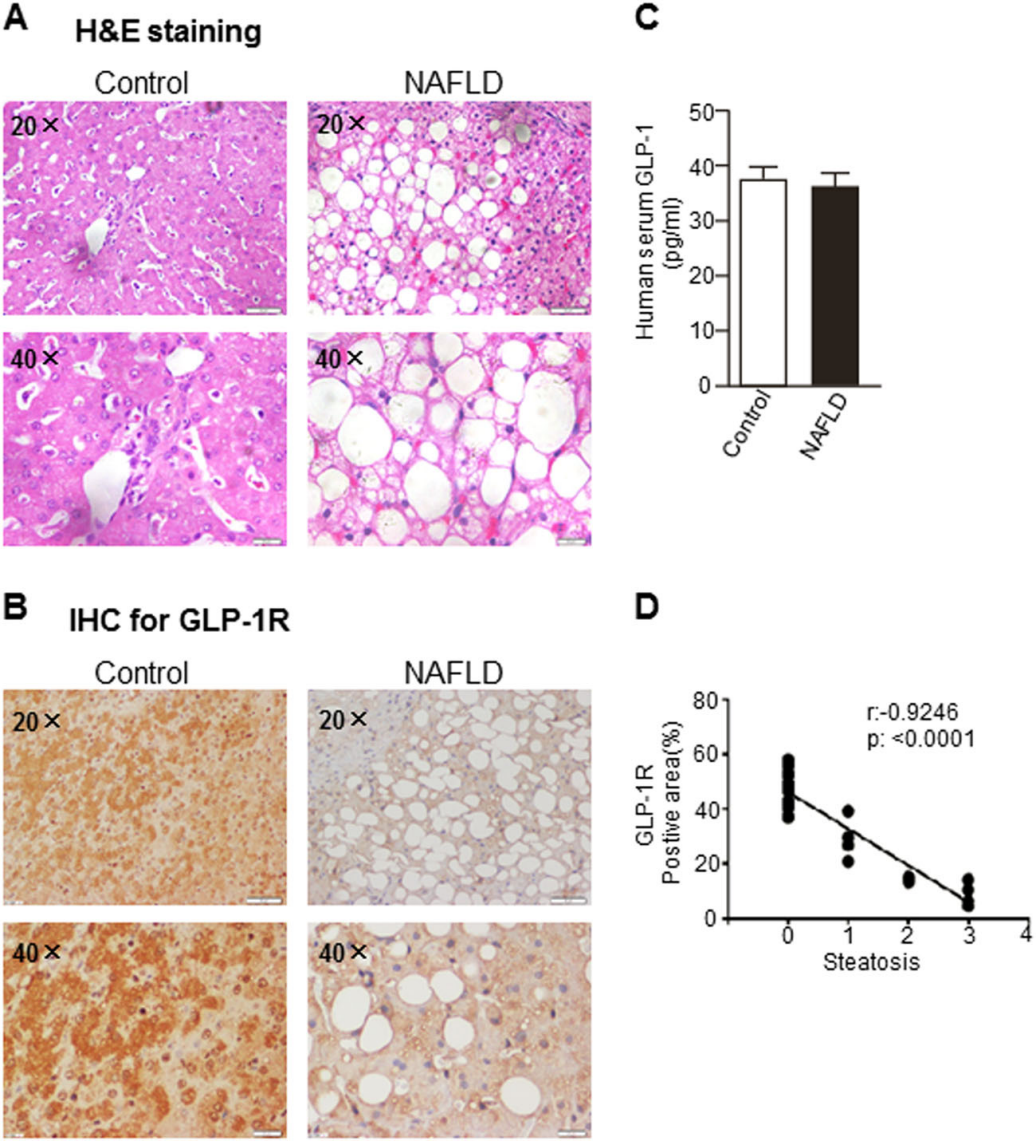
GLP-1R expression levels in the livers of NAFLD patients. **a** Liver HE staining of NAFLD patients. **b** Immunohistochemistry for liver GLP-1R expression in NAFLD patients and normal controls. **c** Serum GLP-1 levels in NAFLD patients (*n* = 40) and normal controls (*n* = 50). **d** The correlation between intrahepatic GLP-1R expression level and hepatic steatosis (*n* = 18). The data represent the means ± S.E.M

Recent clinical studies have shown that GLP-1R agonists represent an attractive treatment option for obese patients with NAFLD^26^. However, the efficacy of GLP-1R agonists in biopsy-proven NASH patients varies, suggesting that the expression levels of GLP-1R may be different in these patients. First, we examined the expression levels of GLP-1R using immunohistochemistry staining in the livers of patients that were diagnosed with NAFLD by liver biopsy and in normal controls. As shown in Fig. 1a, HE staining confirmed the histological diagnosis of NAFLD. The immunohistochemistry showed strong staining of GLP-1R in the livers of normal controls, and it was significantly reduced in livers of NAFLD patients (Fig. 1b). However, ELISA analysis showed that serum GLP-1 levels were similar in normal controls and NAFLD patients (Fig. 1c). A Spearman correlation analysis confirmed that the pathological index of steatosis was negatively correlated with the hepatic GLP-1R expression level (Fig. 1d). Multivariate analysis revealed that the hepatic GLP-1R level was independently associated with hepatic steatosis (Table 1).

**Table 1:** Multiple linear regression analysis of the factors that are involved in GLP-1R expression

Model		Unstandardized coefficients		Standardized coefficients		
		*B*	Std. error	Beta	*t*	Sig.
GLP-1R	Constant	46.508	2.466		18.860	0.000
	Steatosis	−12.881	2.446	−0.887	−5.266	0.000
	Inflammation	−2.263	3.543	−0.117	−0.639	0.533
	Ballooning	1.263	4.042	0.062	0.313	0.759
	Constant	46.566	2.384		19.535	0.000
	Steatosis	−12.529	2.104	−0.863	−5.954	0.000
	Inflammation	−1.624	2.805	−0.084	−0.579	0.571
	Constant	46.216	2.257		20.473	0.000
	Steatosis	−13.432	1.382	−0.925	−9.716	0.000

### NaB prevented the HFD-induced downregulation of GLP-1R in the liver

**Fig. 2:**
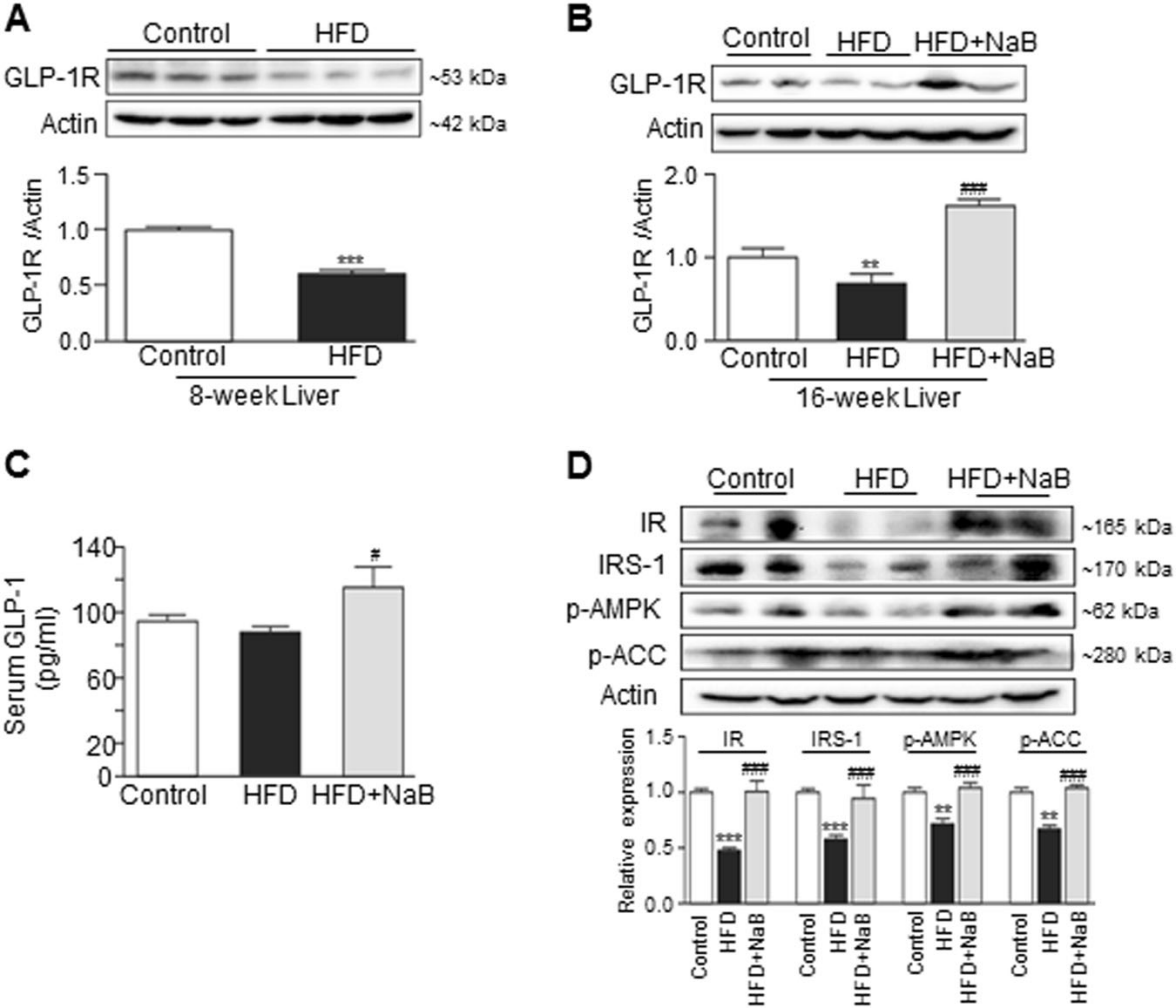
The protein levels of GLP-1R, insulin receptor, IRS-1, p-AMPK, and p-ACC in the livers of HFD-fed mice with or without NaB administration. **a** and **b** Western blot analysis of GLP-1R protein expression in mouse livers after 8 weeks of HFD A, or in mouse livers after 16 weeks of HFD with or without NaB B. **c** Serum levels of GLP-1 in mice after 16 weeks of HFD with or without NaB administration. **d** Western blot analysis of insulin receptor, IRS-1, p-AMPK, and p-ACC protein expression levels in the livers of mice after 16 weeks of HFD with or without NaB administration. The data represent the means ± S.E.M. (*n* = 12 mice per group); vs. control **P < 0.01 and ***P < 0.001; vs. HFD ^#^*P* < 0.05, ^##^*P* < 0.01, and ^###^*P* < 0.001

Our previous study reported that NaB-attenuated HFD-induced steatohepatitis by modulating gut microbiota and intestinal barrier function in mice^10^. To determine whether NaB has any effect on hepatic GLP-1R expression, we first examined the effect of HFD feeding on hepatic GLR-1R expression in the NAFLD mouse model. As shown in Fig. 2a, b, both 8-week and 16-week HFD feeding significantly reduced the GLP-1R protein levels in the mouse livers. NaB treatment reversed the HFD-induced downregulation of GLP-1R after the 8-week treatment (Fig. 2b). Similar to previous findings, NaB also increased serum GLP-1 levels (Fig. 2c). In addition, the Western blot analysis further showed that the HFD-induced suppression of IR, IRS-1, p-AMPK, and p-ACC expression in the liver was also altered by NaB (Fig. 2d).

### NaB improved metabolic indices and prevented disease progression in the HFD-induced NAFLD mouse model

**Fig. 3:**
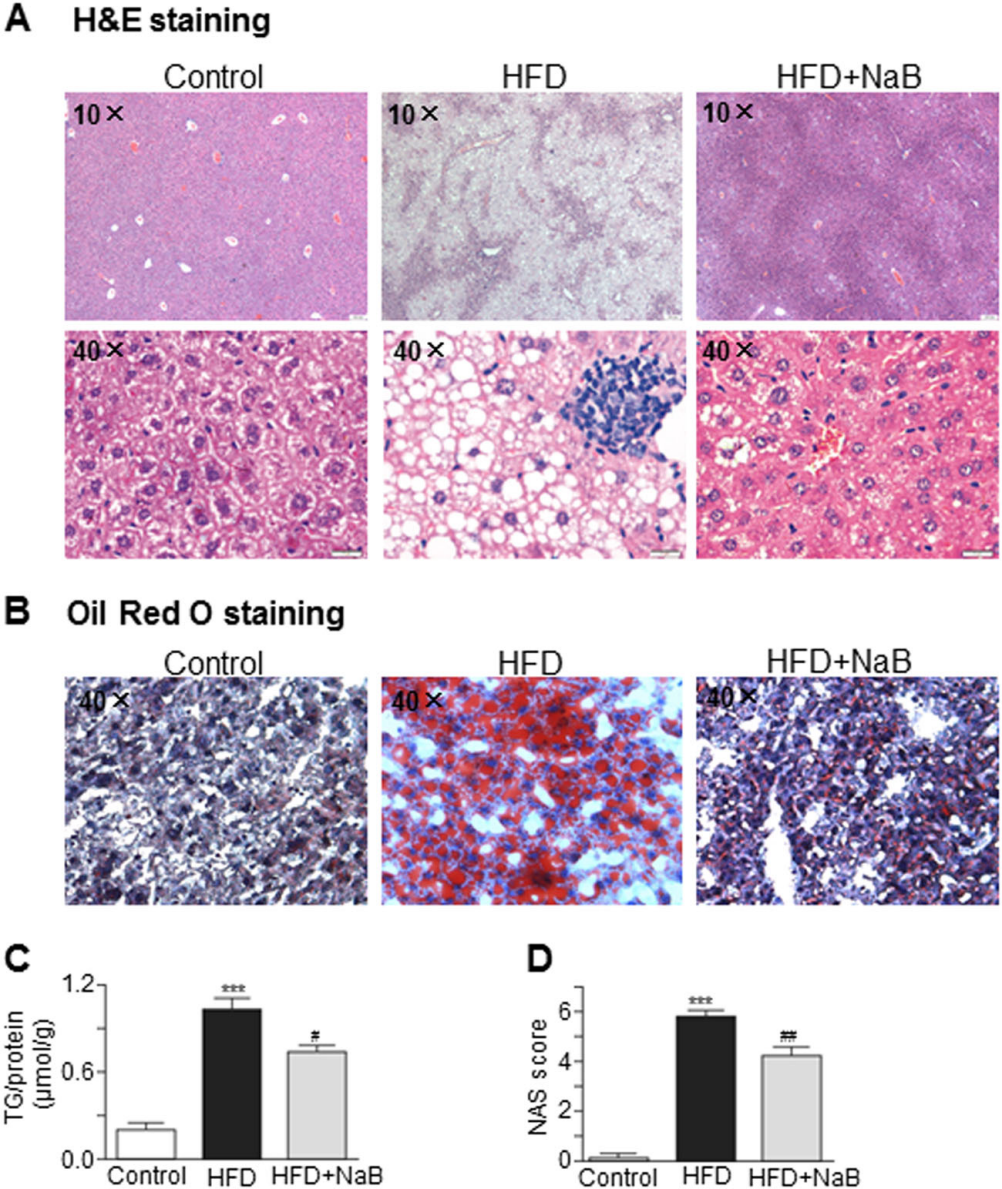
NaB alleviated HFD-induced steatohepatitis in mice. **a** HE staining of mouse livers. **b** Oil Red O staining of mouse livers. **c** Intrahepatic triglyceride concentrations. **d** Liver non-alcoholic fatty liver activity score (NAS). The data represent the means ± S.E.M. (*n* = 12 mice per group); vs. control *P < 0.05, **P < 0.01, and ***P < 0.001; vs. HFD ^#^*P* < 0.05, ^##^*P* < 0.01, and ^###^*P* < 0.001

To determine whether the NaB-mediated upregulation of GLP-1R had a beneficial effect on reducing HFD-induced metabolic stress, we evaluated metabolic indices with or without NaB treatment in HFD-fed mice. As shown in Table 2, the HFD-induced increases in body weight, liver index, fasting serum levels of glucose, ALT and AST were all significantly reduced by treatment with NaB. In addition, NaB intervention improved insulin sensitivity, although the result did not reach statistical significance (Table 2). HE and Oil Red O staining further showed that NaB significantly reduced the HFD-induced hepatic steatosis and lipid accumulation (Fig. 3a, b). The intrahepatic TG levels and NAFLD Activity Score (NAS) were also improved after NaB treatment (Fig. 3c, d).

**Table 2:** Physiological and metabolic parameters

	Control (*n* = 12)	HFD (*n* = 12)	HFD + NaB (*n* = 12)
Body weight (g)	29.33 ± 0.405	37.26 ± 0.833^***^	32.65 ± 0.643^###^
Liver index	4.765 ± 0.056	6.132 ± 0.237^***^	4.801 ± 0.160^###^
Epididymal fat index	1.736 ± 0.122	4.716 ± 0.119^***^	4.352 ± 0.152
Perirenal fat index	0.803 ± 0.081	2.318 ± 0.054^***^	2.264 ± 0.126
Fasting serum glucose (mmol/L)	2.040 ± 0.284	4.262 ± 0.184^**^	2.983 ± 0.572^#^
Fasting serum insulin(ng/mL)	1.916 ± 0.130	1.627 ± 0.080	1.861 ± 0.067
HOMA-IR	3.427 ± 1.096	7.780 ± 0.813^*^	5.788 ± 1.090
ISI	0.023 ± 0.007	0.008 ± 0.002^*^	0.012 ± 0.004
ALT(U/L)	91.53 ± 15.45	361.2 ± 86.36^*^	133.8 ± 11.06^#^
AST(U/L)	178.5 ± 13.65	323.1 ± 18.18^***^	248.0 ± 25.86^#^

vs. control ^*^P < 0.05, ^**^P < 0.01, ^***^P < 0.001; vs. HFD ^#^*P* < 0.05, ^##^*P* < 0.01, ^###^*P* < 0.001

### Effect of FFA and NaB on GLP-1R expression in HepG2 cells

**Fig. 4:**
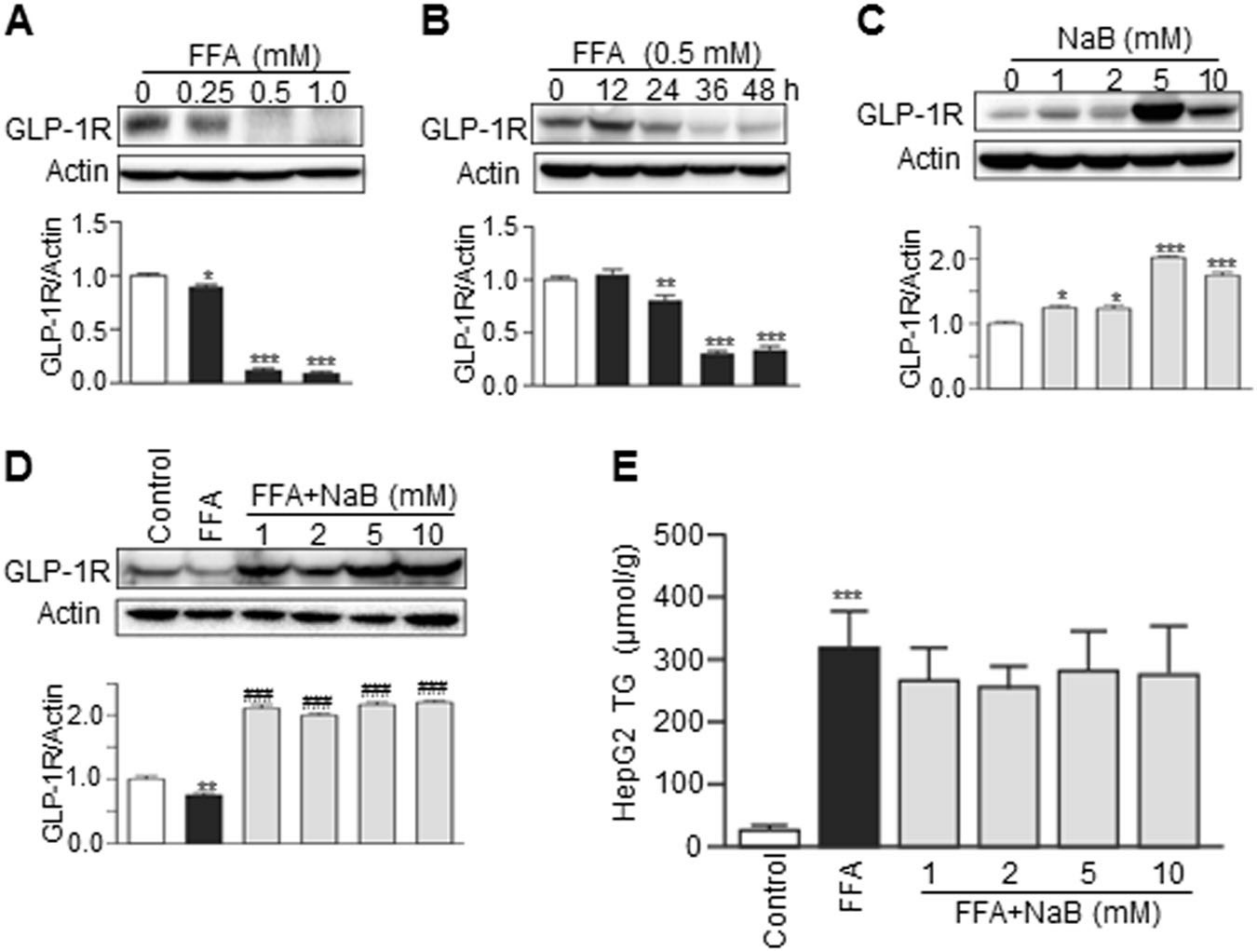
NaB treatment increased GLP-1R protein levels in HepG2 cells with or without FFA treatment. **a–d** Western blot analysis of GLP-1R protein levels in HepG2 cells that were treated with increasing concentrations of FFA for 24 h (**a**), or in HepG2 cells that were treated with FFA for 0 to 48 h (**b**), or in HepG2 cells that were treated with NaB (**c**), or in HepG2 cells that were cotreated with FFA and NaB (**d**). **e** Triglyceride levels in HepG2 cells that were cotreated with FFA (0.5 mM) and NaB (1, 2, 5, or 10 mM). The data represent the means ± S.E.M.; vs. control *P < 0.05, **P < 0.01, and ***P < 0.001; vs. FFA ^##^*P* < 0.01 and ^###^*P* < 0.001

**Fig. 5:**
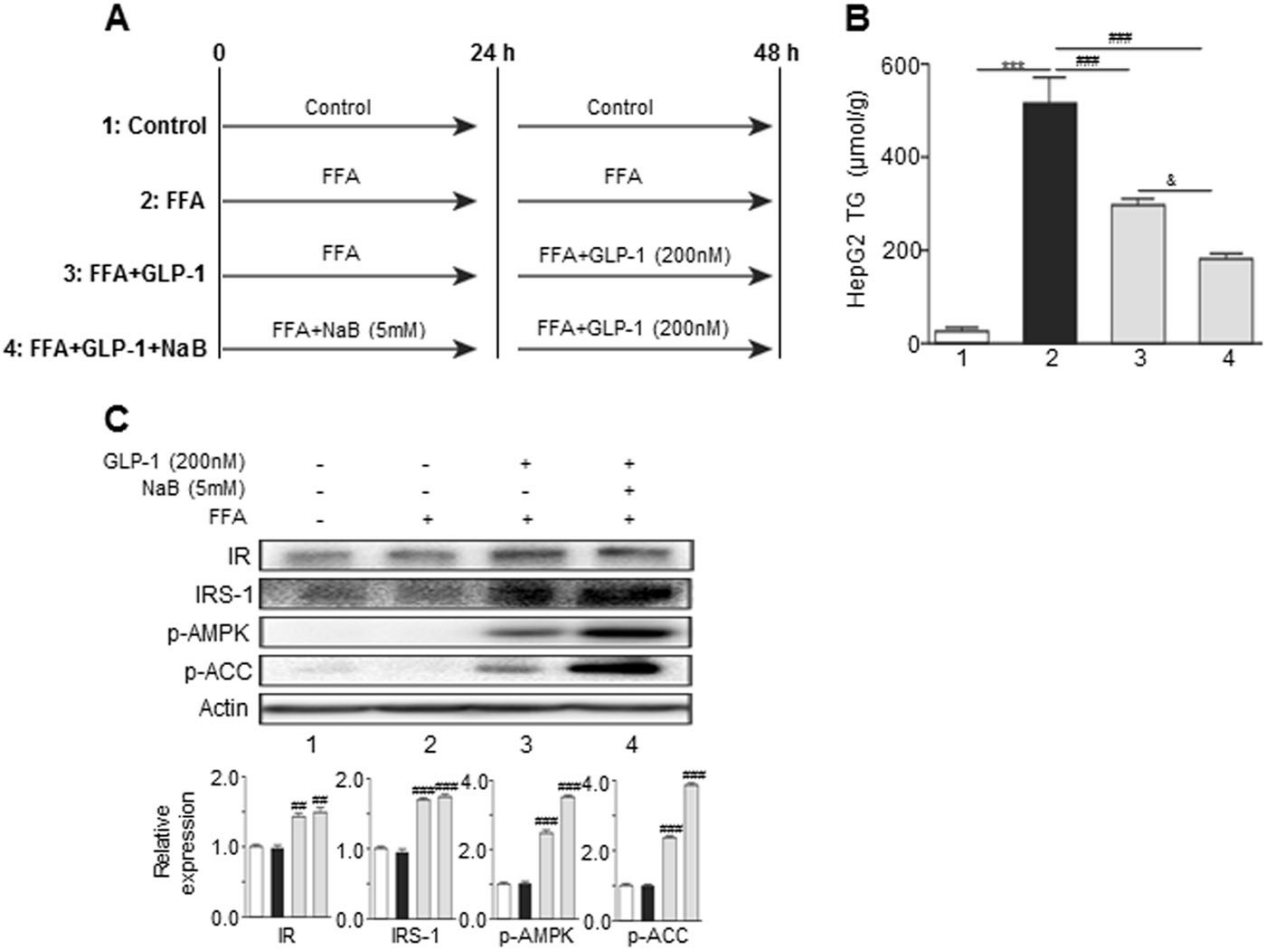
NaB enhanced the effect of GLP-1 on HepG2 cells. **a** Schematic illustration of treatment groups 1, 2, 3, and 4 in the following panels **b** and **c**. **b** Triglyceride levels in HepG2 cells that were treated with the combination of GLP-1 and NaB or treated with GLP-1 alone; treatments of groups 1–4 are shown in panel **a**. **c** Western blot analysis of p-AMPK, p-ACC, insulin receptor, and IRS-1 expression in HepG2 cells that were treated with the combination of GLP-1 and NaB or treated with GLP-1 alone; treatment of groups 1–4 are shown in **a**. The data represent the means ± S.E.M.; vs. control *P < 0.05, **P < 0.01, and ***P < 0.001; vs. FFA ^##^*P* < 0.01 and ^###^*P* < 0.001; ^&^ *P* < 0.05

To further identify the functional mechanism by which NaB prevents HFD-induced hepatic injury, we examined the direct effects of FFAs and NaB on GLP-1R expression in human HepG2 cells. As shown in Fig. 4a, b, FFA inhibited GLP-1R expression in both dose-dependent an`d time-dependent manners. However, NaB dose-dependently upregulated GLP-1R expression (Fig. 4c). In addition, NaB significantly blocked the FFA-induced downregulation of GLP-1R (Fig. 4d). Interestingly, NaB treatment alone (1–10 mM) had no effect on the FFA-induced increase of TG levels in HepG2 cells (Fig. 4e). However, in the presence of GLP-1, NaB potentiated the GLP-1-mediated inhibitory effect on FFA-induced hepatic TG accumulation (Fig. 5a, b). Furthermore, NaB promoted GLP-1-mediated upregulated IRS-1 expression and phosphorylation of AMPK and ACC (Fig. 5c).

### NaB-enhanced hepatic GLP-1R expression via inhibiting HDAC

**Fig. 6:**
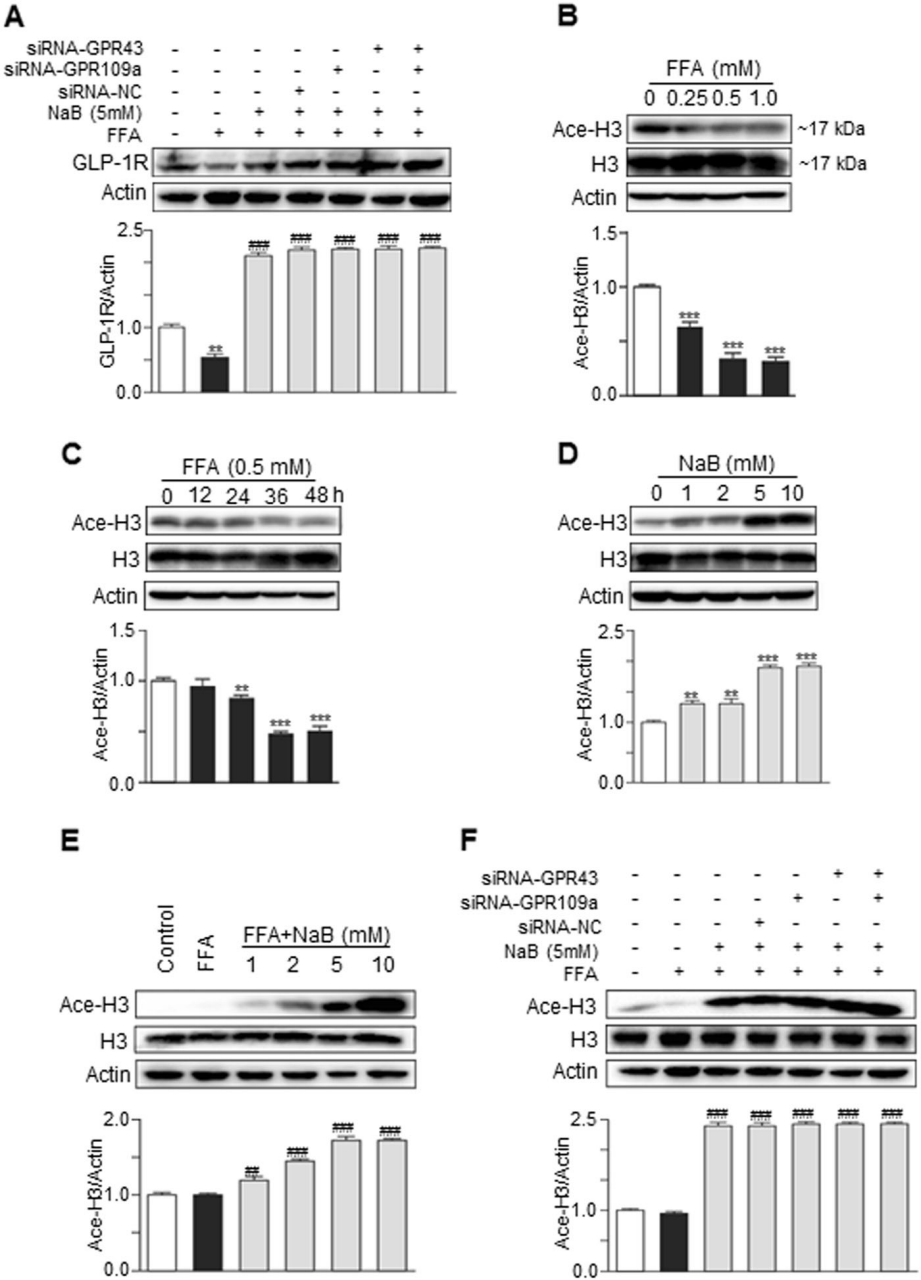
NaB-enhanced GLP-1R expression in HepG2 cells via HDAC inhibition that was independent of GPR43 or GPR109a. **a** Western blot analysis of GLP-1R protein expression in siRNA-transfected HepG2 cells that were cotreated with FFA (0.5 mM) and NaB (5 mM) for 24 h. **b–e** Western blot analysis of total acetyl-histone levels in HepG2 cells that were treated with increasing concentrations of FFA for 24 h (**b**), or in HepG2 cells that were treated with FFA (0.5 mM) for 0 to 48 h (**c**), or in HepG2 cells that were treated with NaB alone (1–10 mM) for 24 h (**d**), or in HepG2 cells that were cotreated with FFA (0.5 mM) and NaB for 24 h (**e**). **f** Western blot analysis of total acetyl-histone levels in siRNA-transfected HepG2 cells that were cotreated with FFA (0.5 mM) and NaB. The data represent the means ± S.E.M.; vs. control **P < 0.01 and ***P < 0.001; vs. FFA ^##^*P* < 0.01 and ^###^*P* < 0.001

It has been reported that short-chain fatty acids (SCFAs) including NaB are able to regulate the immune response and metabolism through the activation of GPRs, such as GRP43 and GRP109a or through the inhibition of HDAC^27,28^. To elucidate the possible cellular mechanisms of NaB-mediated upregulation of GLP-1R expression in hepatocytes, gene-specific siRNAs were used to silence GRP43 and GRP109a. As shown in Fig. 6a, the NaB-mediated inhibition of the FFA-induced downregulation of GLP-1R was not affected by GRP43 or GRP109a siRNAs. However, FFA inhibited histone 3 acetylation in dose-dependent and time-dependent manners in hepatocytes (Fig. 6b, c). In contrast, NaB dose-dependently increased histone 3 acetylation both in the absence and presence of FFA (Fig. 6d, e). Furthermore, the NaB-induced upregulation of histone 3 acetylation was not affected by either GRP43 siRNA or GRP109a siRNA (Fig. 6f) in HepG2 cells.

**Fig. 7:**
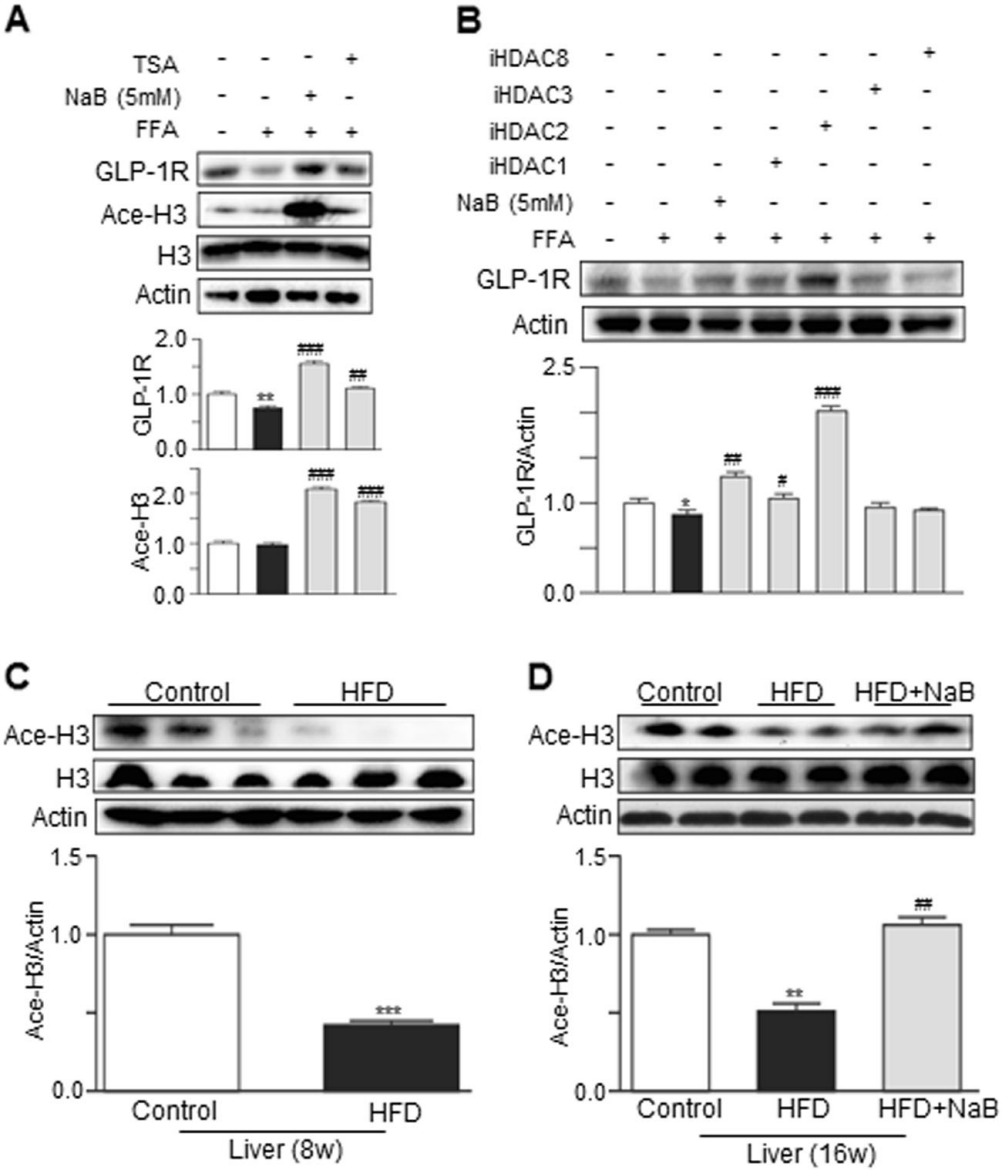
The GLP-1R expression in HepG2 was primarily regulated by HDAC2, and the acetyl-histone protein levels in the liver were decreased after 8 or 16 weeks of HFD but were upregulated after NaB intervention. **a** Western blot analysis of the total acetyl-histone and GLP-1R levels in HepG2 cells that were cotreated with FFA (0.5 mM) and NaB or cotreated with FFA and TSA. **b** Western blot analysis of the GLP-1R levels in HepG2 cells that were cotreated with FFA and an HDAC1 inhibitor, HDAC2 inhibitor, HDAC3 inhibitor, or HDAC8 inhibitor, respectively. **c** Western blot analysis of the total acetyl-histone protein levels in mouse livers after 8 weeks on the HFD. **d** Western blot analysis of the total acetyl-histone protein levels in mouse livers after 16 weeks on the HFD with or without NaB administration. The data represent the means ± S.E.M.; vs. control **P < 0.01 and ***P < 0.001; vs. FFA or HFD ^##^*P* < 0.01 and ^###^*P* < 0.001

To confirm the association between histone acetylation and GLP-1R expression, we used a universal HDAC inhibitor (TSA). As shown in Fig. 7a, TSA also reversed the FFA-induced downregulation of GLP-1R and acetyl-H3 in HepG2 cells. To further identify the specific HDAC isoform that was involved in regulating GLP-1R expression, isoform-specific HDAC inhibitors were used. As shown in Fig. 7b, iHDAC2 showed the most profound effect on GLP-1R expression in HepG2 cells. Consistent with our findings in HepG2 cells, the in vivo studies also showed that HFD feeding markedly inhibited the acetylation of histone 3, and this effect was reversed by NaB treatment (Fig. 7c, d).

## Discussion

**Fig. 8:**
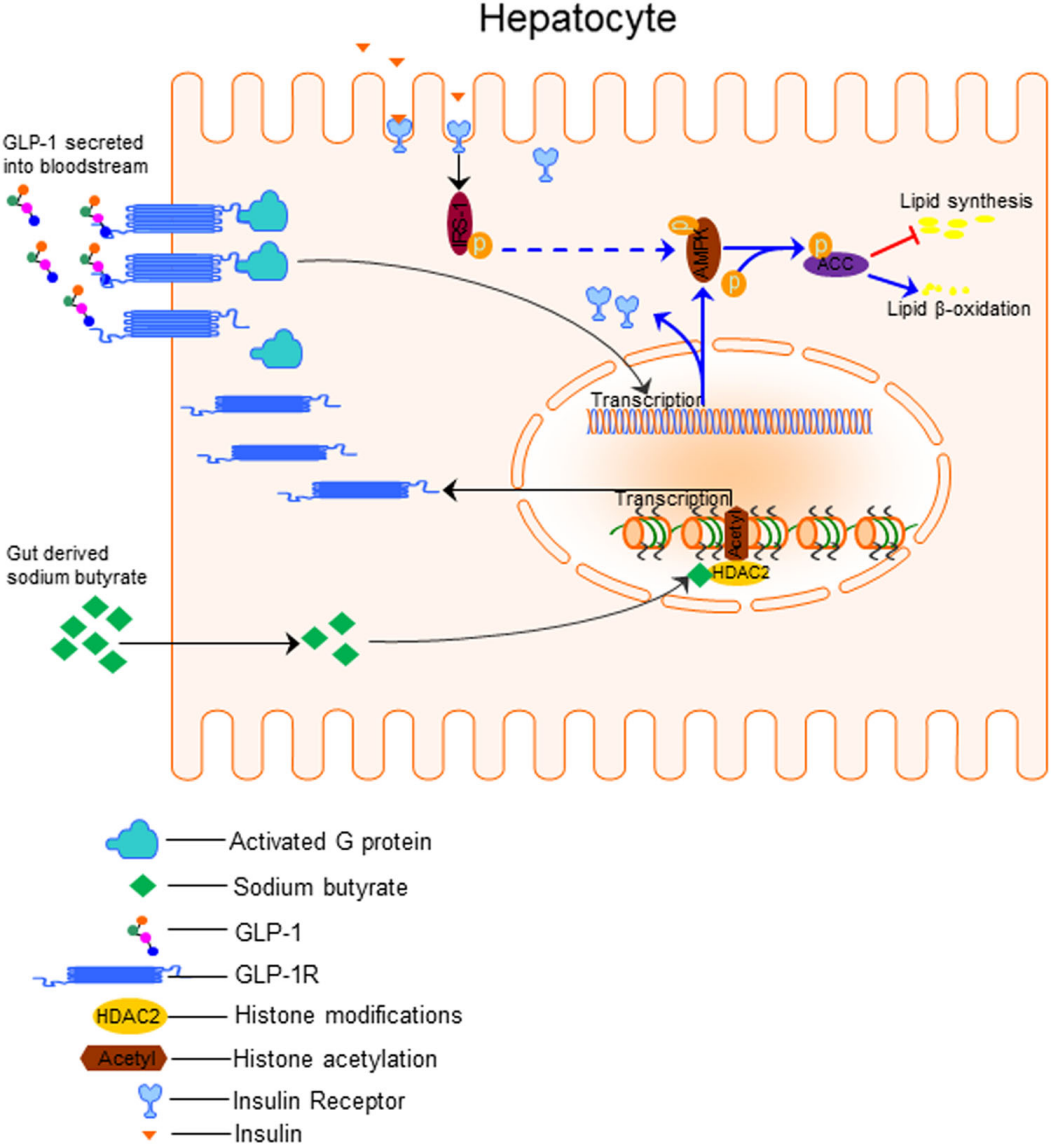
Schematic illustration of the mechanism employed by NaB in improving liver GLP-1 resistance

Here, we identified the novel potential mechanisms underlying NaB-mediated beneficial effects in preventing NAFL disease progression. The lipotoxicity of FFA contributed to the downregulation of hepatic GLP-1R, which led to hepatic unresponsiveness to GLP-1 and disease progression. Our study demonstrated that NaB upregulated hepatic GLP-1R expression by inhibiting HDAC2, which improved hepatic GLP-1 sensitivity and prevented NAFL disease progression (Fig. 8).

NASH remains a complex, multifaceted issue with no standard treatment that is currently available^3^. Studies have shown positive results indicating that GLP-1 or its analog can reduce hepatic gluconeogenesis and lipogenesis, suggesting that GLP-1 can be used in the treatment of NASH^29–31^. However, several clinical studies have shown that GLP-1 intervention did not improve hepatic steatosis in NAFLD patients^18,19^. GLP-1 resistance has recently been reported in T2D patients^20^. Our results showed that both FFA and HFD induced the loss of GLP-1 responsiveness in in vitro-cultured hepatocytes and in the in vivo animal model, which supported our central hypothesis. The loss of hepatic GLP-1 responsiveness in NAFLD patients represents an important mechanism of disease progression of NASH. Therefore, a GLP-1 sensitizer will have a beneficial effect in preventing NASH.

It has been reported that the dysbiosis of the gut microbiota contributed to the occurrence of GLP-1 resistance in T2D mice. Additionally, both the phylum Firmicutes and the genus *Lactobacilli* were positively correlated with GLP-1R levels in the ileum, indicating that the molecular mechanisms that are responsible for GLP-1 resistance in HFD-fed mice could be primarily related to reduced GLP-1R expression^20^. Of note, our previous work demonstrated that the gut metabolite NaB markedly reversed HFD-induced dysbiosis of the gut microbiota in mice and significantly elevated the abundance of *Lactobacilli* in the gut^10^, which supported the hypothesis that NaB might have the potential to improve GLP-1 resistance.

It is well-known that functional GLP-1R is indispensable for GLP-1 action^13^. Existing evidence has indicated that liver GLP-1R expression is significantly decreased in NASH patients^15^. In the current study, we confirmed that liver GLP-1R expression was downregulated in NAFLD patients compared with its expression in normal controls. In addition, we found that the lower GLP-1R expression is associated with the severity of hepatic steatosis. In the NAFLD animal model, hepatic GLP-1R expression levels at the NAFL (8 weeks) and NASH (16 weeks) stages were downregulated, consistent with the data in humans. These findings suggested that the therapeutic effects of GLP-1 are limited in NAFLD patients with lower GLP-1R expressions. Therefore, it will be worthwhile and meaningful to explore a “GLP-1 sensitizer” to enhance the hepatic responsiveness to GLP-1. Our previous study demonstrated that NaB significantly improved hepatic steatosis^10^. In the current study, we further showed that NaB alleviated hepatic steatosis via enhancing hepatic GLP-1 sensitivity by GLP-1R expression.

The gut microbiota is a regulatory endocrine organ, and it plays a critical role in regulating lipid, glucose, and energy metabolism. Gut microbiota-derived SCFAs are important players in the maintenance of metabolic homeostasis^32–36^. Our results showed that the serum GLP-1 levels were significantly elevated after NaB treatment, and these findings were supported by previous studies showing that NaB has the ability to promote GLP-1 secretion from enteroendocrine L cells^8,12,37^. In addition, NaB treatment induced GLP-1R expression in human HepG2 cells and markedly reversed the FFA-induced downregulation of GLP-1R expression but not the expression of TNF-α or lipopolysaccharide in vitro (Fig. S1). Similarly, NaB prevented the HFD-induced downregulation of GLP-1R in vivo. In addition, we found that TG levels in HepG2 cells were not reduced by NaB intervention alone but were markedly reduced by the combination of NaB with GLP-1, suggesting that NaB alleviated hepatic steatosis partially through enhancing GLP-1 action.

It is well-established that disruption of the hepatic fatty acid oxidation, lipogenesis, and insulin signaling pathways contributes to hepatic steatosis. Previous studies have demonstrated that the cAMP/p-AMPK/p-ACC and insulin-sensitizing pathways are pivotal to GLP-1’s beneficial effects on hepatic lipid metabolism. Previously, p-AMPK was identified as a vital regulator of hepatic lipid metabolism which promotes lipid β-oxidation and inhibits lipid synthesis via inducing ACC phosphorylation^13,14^. In this study, we found that NaB and GLP-1 additively induced the p-AMPK/p-ACC and insulin receptor/IRS-1 pathways both in HepG2 cells and in the in vivo NAFLD mouse model. These results suggest that NaB-induced hepatic GLP-1R expression is critical for improving the GLP-1’s therapeutic effects.

It has been shown that NaB can activate the cell surface receptors GPR43 or GPR109a to mediate its physiological functions (Fig. S2)^12^. However, the results of the current study indicated that the NaB-mediated upregulation of GLP-1R expression is independent of GPR43 or GPR109a. Considering the previously reported effect of NaB on class I HDACs^38,39^, we further examined the role of HDACs in NaB-mediated GLP-1 expression. Of note, we found that the decrease in GLP-1R expression in steatotic hepatocytes was significantly associated with the downregulation of histone H3 acetylation that was induced by FFAs. In contrast, the total H3 acetylation in the liver was significantly enhanced after NaB intervention in vivo, and the total H3 acetylation was also obviously enhanced in vitro in HepG2 cells after NaB treatment, independent of GPR43 or GPR109a. Furthermore, TSA (a nonspecific HDAC inhibitor) treatment successfully mimicked the effect of NaB on GLP-1R expression in HepG2 cells, and this finding strongly supported the idea that NaB may affect liver GLP-1 sensitivity through HDAC inhibition. To further evaluate which specific HDAC inhibition recapitulated this enhancement of GLP-1R expression in hepatocytes, we used four types of specific HDAC inhibitors, which all belonged to class I HDACs. The results demonstrated that HDAC2 inhibition, rather than HDAC1, HDAC3, or HDAC8 inhibition, predominantly mimicked the effect of NaB on increasing GLP-1R expression in HepG2 cells. HDAC1, 2, and 3 share structural and functional similarity. The effects of the HDAC1 and HDAC3 inhibitors on GLP-1R expression might be associated with a weak inhibition of HDAC2 due to cross-reactivity of these inhibitors. These results suggested a critical role for HDAC2 inhibition in this process, but the exact transcription factors still need to be determined.

In conclusion, the current study suggests that loss of hepatic GLP-1R in NAFLD patients is responsible for low responsiveness to GLP-1 therapy. The combinational therapy of NaB with GLP-1 has great potential to alleviate NAFL and NASH. These findings indicate that patients who are undergoing GLP-1 therapy may further benefit from this treatment by consuming more dietary fiber, which can be transformed into butyrate in the gut to enhance the beneficial effects of GLP-1. The current study has conceptual and practical implications for the initiation of future clinical studies of NAFLD.

## Electronic supplementary material

Supplementary Data
