# Supplementary material for: Sodium butyrate reduces high-fat diet-induced non-alcoholic steatohepatitis through upregulation of hepatic GLP-1R expression
Source: Exp Mol Med. 2018 Dec 3;50(12):157. doi: 10.1038/s12276-018-0183-1 (PMC6277380; doi:10.1038/s12276-018-0183-1)
Supplement: Supplementary file 1 — Supplementary Data [file 12276_2018_183_MOESM1_ESM.doc]

**Title: Sodium butyrate reduces high fat diet-induced non-alcoholic steatohepatitis through upregulation of hepatic GLP-1R expression**

**Table of contents**

Supplementary material………………………………..…………………….2

Figure S1....…..…………..……………………………………..………….….3

Figure S2...……………………………………...…………............................3

Table S1………………………………………………….…..…………………4

**Supplementary materials**

*Antibodies and reagents:* CI-994 (HY-50934, HDAC1 inhibitor, 5 μM), Santacruzamate A (HY-N0931, HDAC2 inhibitor, 1 nM), RGFP966 ([HY-13909](http://www.medchemexpress.cn/rgfp966.html), HDAC3 inhibitor, 1 μM), PCI-34051 ([HY-15224](http://www.medchemexpress.cn/pci-34051.html), HDAC8 inhibitor, 100 nM) and Trichostatin A ([HY-15144](http://www.medchemexpress.cn/trichostatin-a.html), TSA, nonspecific HDAC inhibitor, 10 nM) were purchased from MedChem Express (USA) and used in our study. Sodium butyrate (303410), lipopolysaccharides (LPS, L6529), palmitic acid (P5585), oleic acid (O1383) were purchased from Sigma-Aldrich (St. Louis, MO, USA). Tumor necrosis factor-α (TNF-α) was purchased from Peprotech (300-01, USA).

**
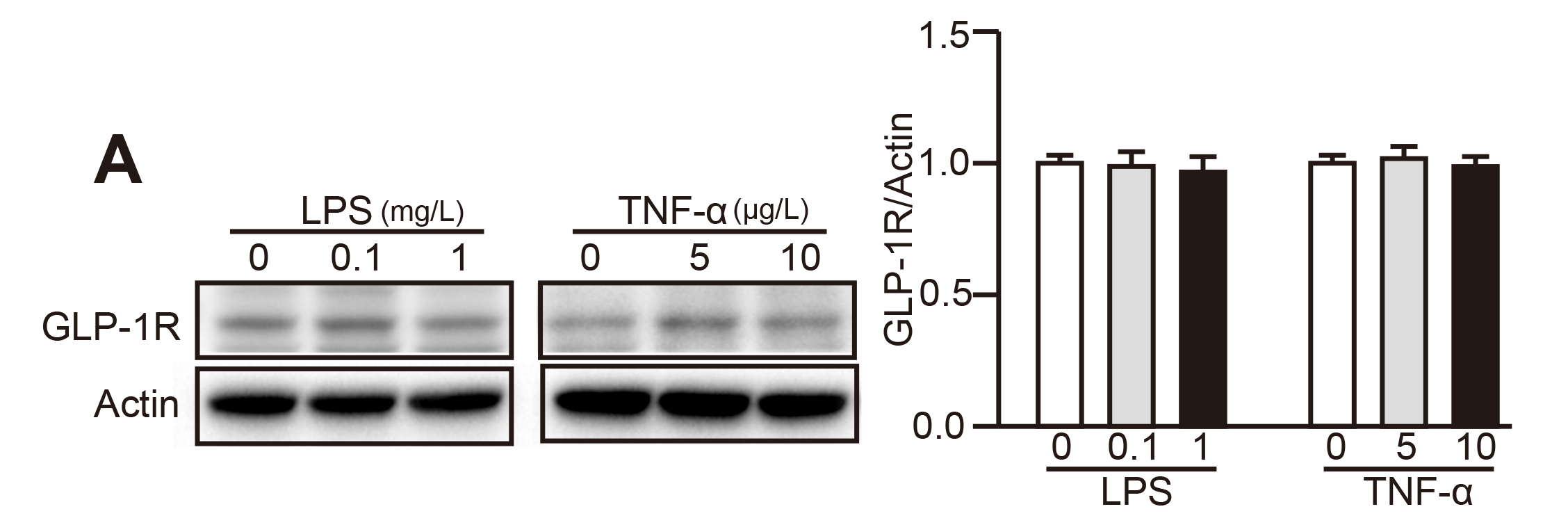
**

**Figure S1. The effects of lipopolysaccharides or TNF-α treatment on GLP-1R protein levels in HepG2 cells.** **A.** Western blot analysis of GLP-1R protein levels in HepG2 cells co-treated with LPS or TNF-α for 24 hours.


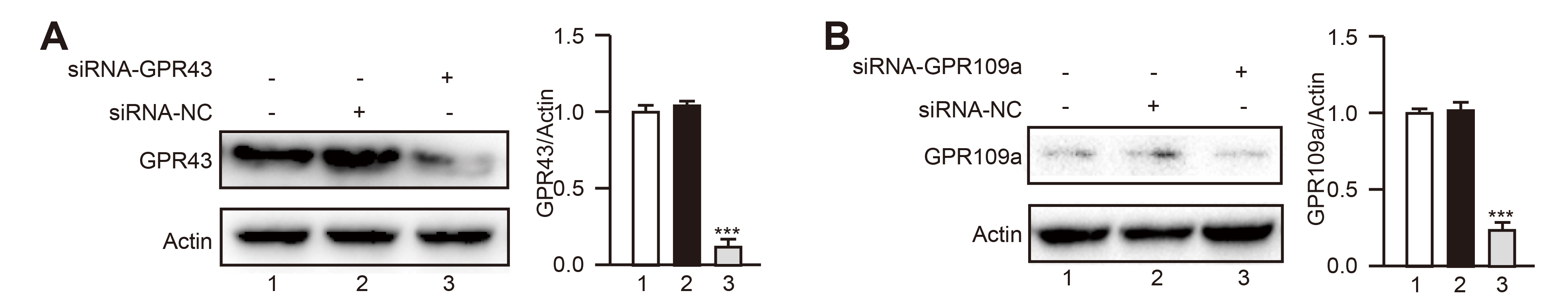


**Figure S2. GPR43 or GPR109a expression in HepG2 cells was successfully knocked out by siRNA.** **A.** GPR43 protein expression in HepG2 cells was significantly down-regulated by siRNA-GPR43 when compared with the normal control. **B.** GPR109a protein expression in HepG2 cells was significantly down-regulated by siRNA-GPR109a when compared with the normal control. The data represent the mean ± S.E.M. vs. control **P* < 0.05, ***P* < 0.01 and ****P* < 0.001.

**Table S1** List of siRNA sequences

| **Gene** | **Forward sequence** | **Reverse sequence** |
| --- | --- | --- |
| GPR43 | GGUCAAUAGCCGUGGUGUUTT | AACACCACGGCUAUUGACCTT |
| GPR109a | CCAUCUGCAGGAUCACUUUTT | AAAGUGAUCCUGCAGAUGGTT |
